# Supplementary material for: Sry-modified laboratory rat lines to study sex-chromosome effects underlying sex differences in physiology and disease: Four Core Genotypes and more
Source: Biol Sex Differ. 2026 Feb 21;17:75. doi: 10.1186/s13293-026-00837-5 (PMC13067745; doi:10.1186/s13293-026-00837-5)
Supplement: Supplementary file 1 — Supplementary Material 1 [file 13293_2026_837_MOESM1_ESM.docx]

**Supplementary Figure 1**. Alignment of long-read sequence to laboratory host *E. coli* bacterial genomic DNA. Some *E. coli* genomic DNA copurified with the BAC prior to microinjection is co-inserted at each integration site within each transgenic line. Aligned regions ranged from ~2.4- to ~68-kbp.


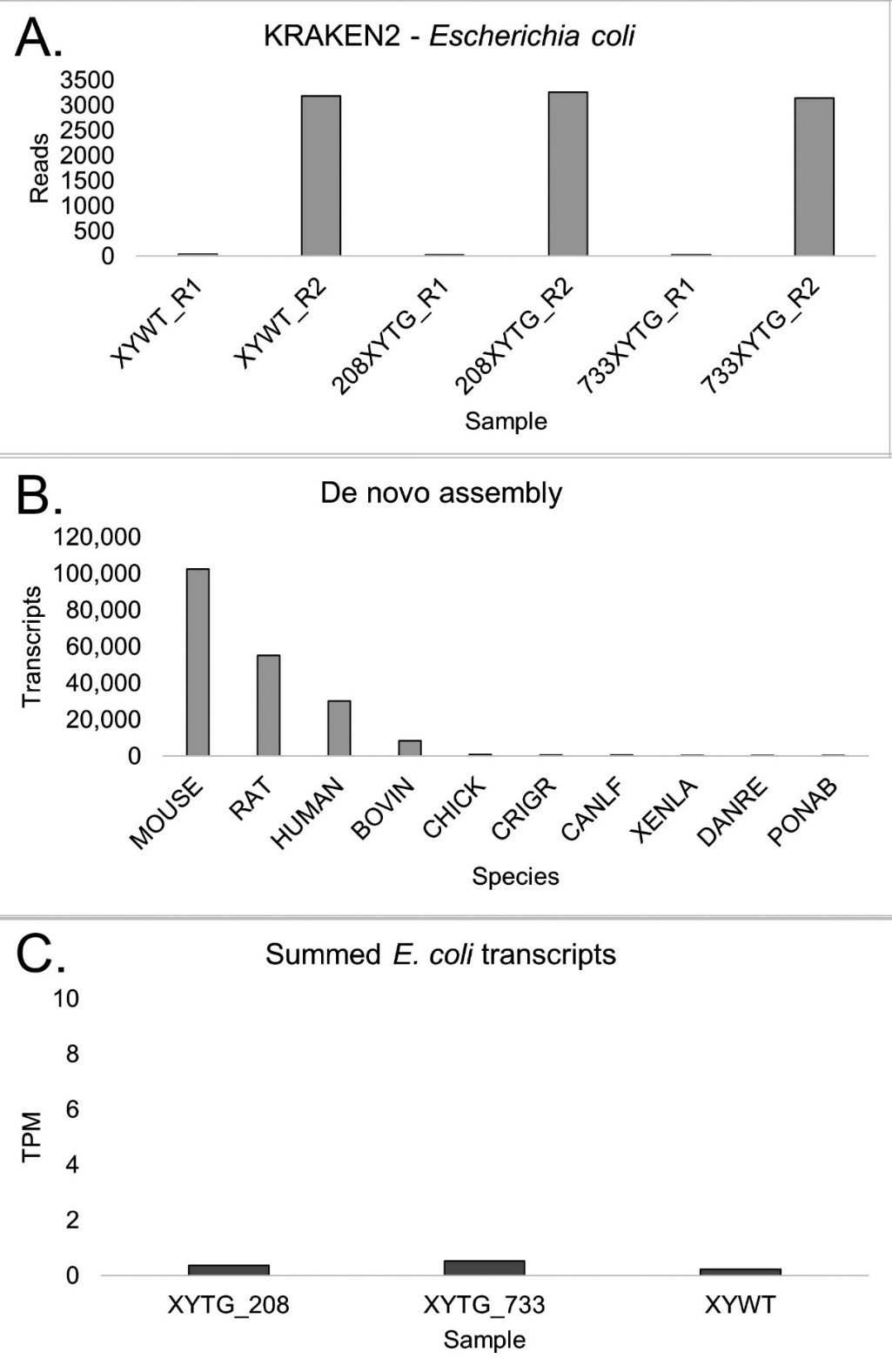
**Supplementary Figure 2:** Testing for expression of *E. coli* transcripts

A. The number of reads mapping to *E coli* genome from either end (R1 or R2) of liver RNAseq per sample. The read numbers were similar in two transgenic lines 208 and 733, which contain some *E. coli* genome, relative to an XYWT male, which lacks any E. coli genome, indicating that read numbers were at background in each case. B. Number of transcripts per species from de novo assembly. BOVIN, bovine; CRIGR, *Cricetulus griseus* (Chinese hamster); CANLF, *Canis lupus familiaris* (dog); XENLA, *Xenopus laevis*; DANRE, *Danio rerio;* PONAB, *Pongo abelii* (Sumatran orangutan). C. Transcripts per million (TPM) of the three *de novo* constructed *E. coli* transcripts were all below 1 TPM background noise.
